# Localized extension in megathrust hanging wall following great earthquakes in western Nepal

**DOI:** 10.1038/s41598-021-00297-4

**Published:** 2021-11-02

**Authors:** Magali Riesner, Laurent Bollinger, Judith Hubbard, Cyrielle Guérin, Marthe Lefèvre, Amaury Vallage, Chanda Basnet Shah, Thakur Prasad Kandel, Samuel Haines, Soma Nath Sapkota

**Affiliations:** 1 Earth Observatory of Singapore, NTU, Singapore, Singapore; 2 CEA, DAM, DIF, 91297 Arpajon, France; 3 Institut de Physique du Globe de Paris, Paris, France; 4 Department of Mines and Geology, Kathmandu, Nepal

**Keywords:** Natural hazards, Solid Earth sciences

## Abstract

The largest (M8+) known earthquakes in the Himalaya have ruptured the upper locked section of the Main Himalayan Thrust zone, offsetting the ground surface along the Main Frontal Thrust at the range front. However, out-of-sequence active structures have received less attention. One of the most impressive examples of such faults is the active fault that generally follows the surface trace of the Main Boundary Thrust (MBT). This fault has generated a clear geomorphological signature of recent deformation in eastern and western Nepal, as well as further west in India. We focus on western Nepal, between the municipalities of Surkhet and Gorahi where this fault is well expressed. Although the fault system as a whole is accommodating contraction, across most of its length, this particular fault appears geomorphologically as a normal fault, indicating crustal extension in the hanging wall of the MHT. We focus this study on the reactivation of the MBT along the Surkhet-Gorahi segment of the surface trace of the newly named Reactivated Boundary Fault, which is ~ 120 km long. We first generate a high-resolution Digital Elevation Model from triplets of high-resolution Pleiades images and use this to map the fault scarp and its geomorphological lateral variation. For most of its length, normal motion slip is observed with a dip varying between 20° and 60° and a maximum cumulative vertical offset of 27 m. We then present evidence for recent normal faulting in a trench located in the village of Sukhetal. Radiocarbon dating of detrital charcoals sampled in the hanging wall of the fault, including the main colluvial wedge and overlying sedimentary layers, suggest that the last event occurred in the early sixteenth century. This period saw the devastating 1505 earthquake, which produced ~ 23 m of slip on the Main Frontal Thrust. Linked or not, the ruptures on the MFT and MBT happened within a short time period compared to the centuries of quiescence of the faults that followed. We suggest that episodic normal-sense activity of the MBT could be related to large earthquakes rupturing the MFT, given its proximity, the sense of motion, and the large distance that separates the MBT from the downdip end of the locked fault zone of the MHT fault system. We discuss these results and their implications for the frontal Himalayan thrust system.

## Introduction

The largest earthquakes on megathrust fault systems propagate from the brittle-ductile transition zone to the surface, rupturing the ground along the frontal fault^1–3^. Such large earthquakes can also cause significant off-fault deformation during the co- and post-seismic periods due to the large stress changes induced by the main slip event, observed as slip on secondary faults within the hanging wall (e.g. following the 1905 Kangra and 2011 Tohoku-Oki earthquakes^4,5^). Notably, this reactivation may illuminate a different stress regime, as the changes in crustal stress state can cause a temporary rotation of the principal stress directions (e.g.^6,7^). One of the most compelling examples of this process is the association of crustal extension several tens of kilometers away from the front with large megathrust earthquakes (e.g.^8^). This linkage has been documented by observation of a landward-dipping normal fault that slipped during or soon after the 2011 Tohoku-Oki earthquake^5^, and a similar landward-dipping normal fault has been identified in the Shumagin Gap of the Alaska subduction zone^9^. However, these subduction settings do not allow the collection of paleoseismic data to document the past history of the fault. Identifying this process in onshore settings would provide a means to extend the past history of this process, and better understand how the slip history of such normal faults is related to megathrust earthquakes.

**Figure 1:**
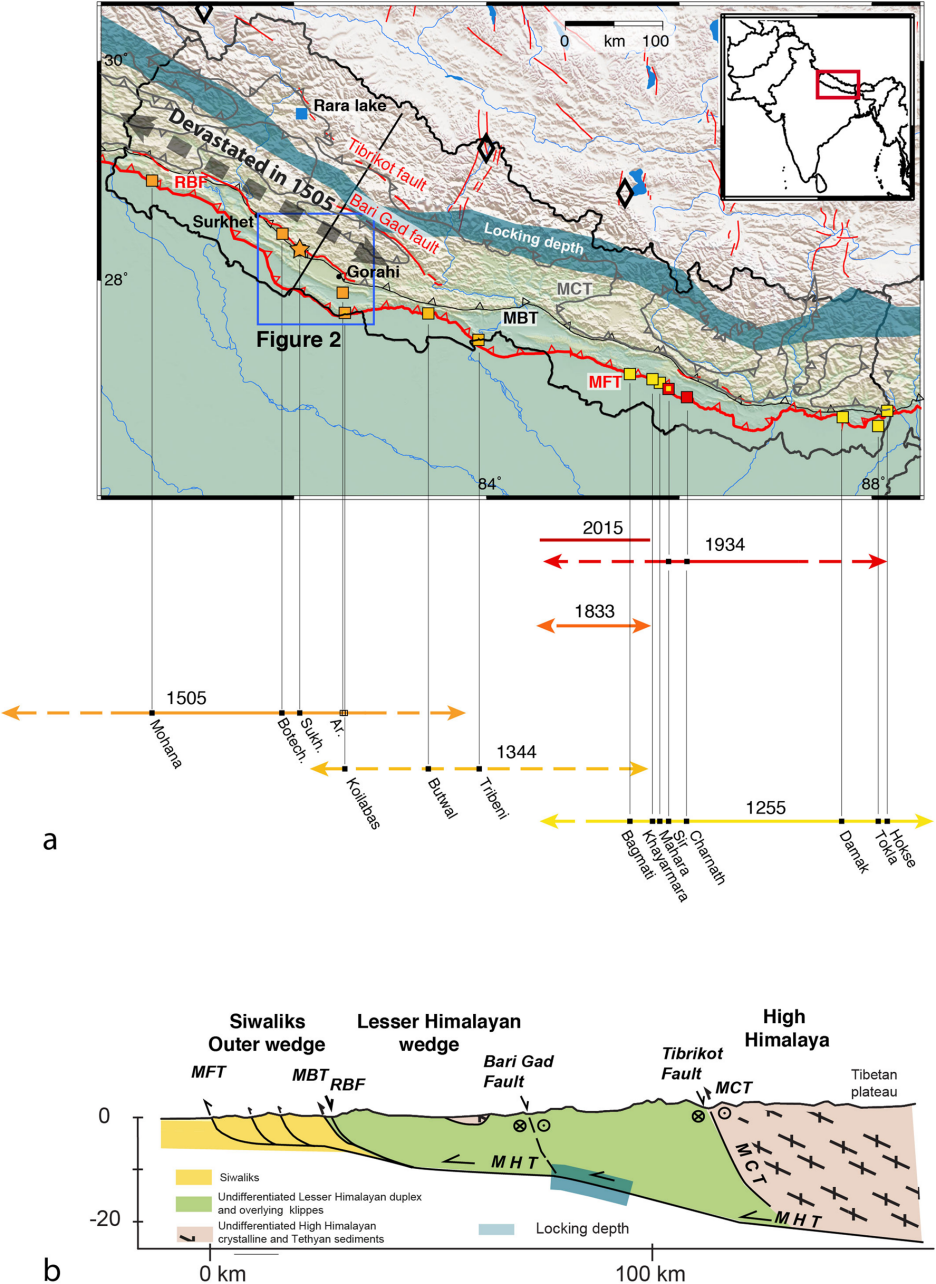
(**a**) Tectonic map of Nepal with active faults in red and locking line in blue (after^2^). Rupture lengths and return time of great Himalayan earthquakes in Nepal since 1223 AD. Rupture extent are consistent with limited macroseismic historical evidence and growing paleoseismological and morphological data. Colour and black squares respectively correspond to the location of the paleoseimic sites excavated along strike and the Rara lake^27–34,40^. Blue box locates the frame of Fig. 2. The star represents the Sukhetal site excavated during the November 2018 fieldtrip. Black lines highlight cross-sections of (**b**) and Fig. 9. (**b**) Tectonic sketch of the main structures across Nepal. MFT: Main Frontal Thrust, MBT: Main Boundary Thrust, RBF: Reactivated Boundary Fault. Figure generated with Adobe illustrator CS6 (http://www.adobe.com/fr/products/illustrator.html).

Similarly, in the Himalayas, some active relief-ward dipping normal faults have been observed^10–19^. Among them, there are reactivated segments of the Main Boundary Thrust system (hereafter called the Reactivated Boundary Fault—RBF—Fig. 1) that may be the onshore analog of landward-dipping normal faults observed in accretionary wedges slipping in association with large earthquakes.

**Figure 2:**
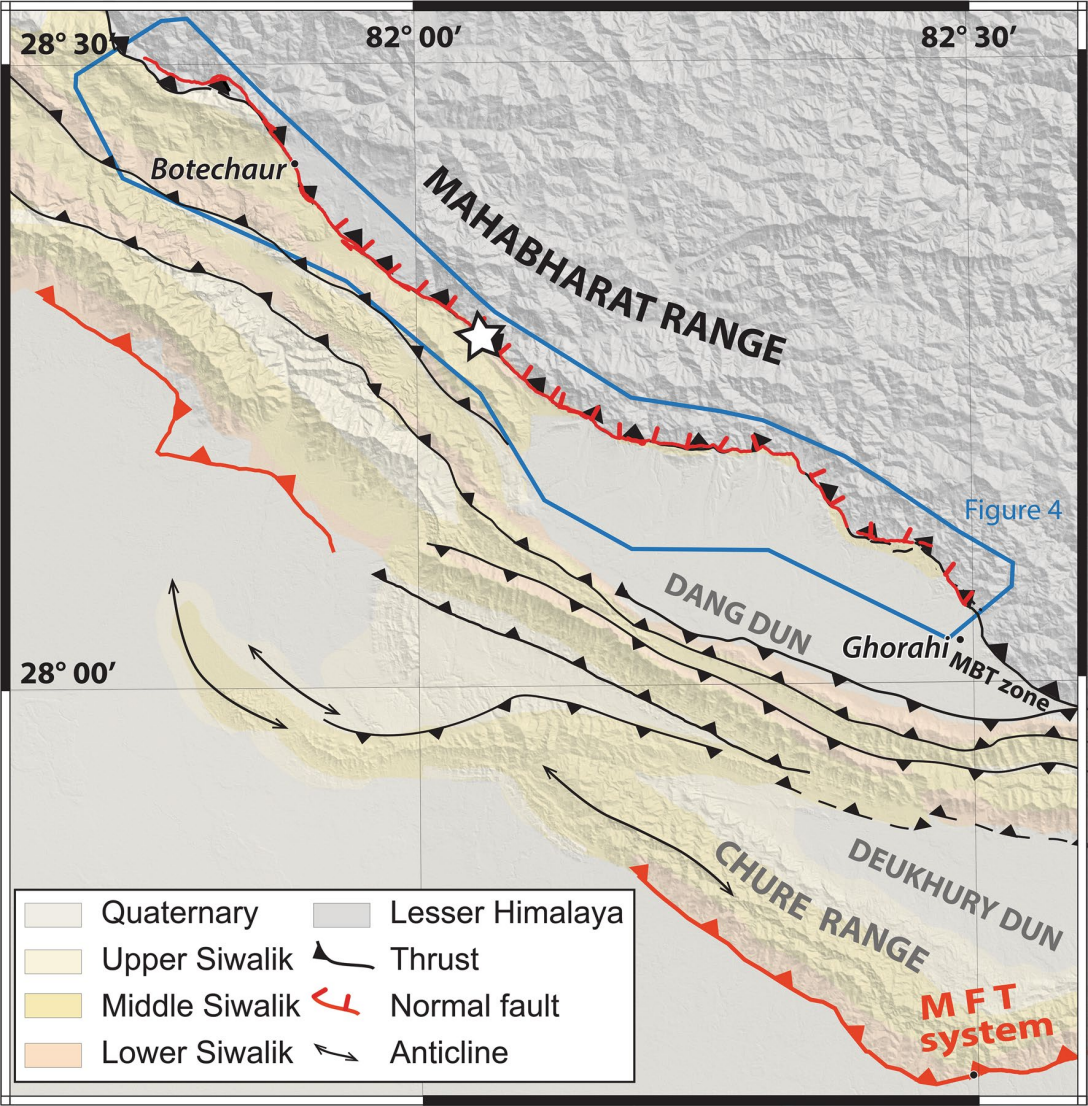
Regional structural and geological map of the frontal Himalayan thrust system in western Nepal compiled from Dhital^19^; Mugnier et al.^41^; the Geological exploration map of Nepal by the Petroleum Exploration Promotion Project of the Department of Mines and Geology, Nepal and mapping from satellite images. Frame located on Fig. 1. Blue box locates our high-resolution Pléiades images Fig. 4. The Surkhet-Gorahi segment of the Reactivated Boundary Fault (reactivated Main Boundary Thrust) is represented by red lines. MFT: Main Frontal Thrust. (1) Highlights the Sukhetal site where a pit and a trench were excavated. Dang and Deukhury Dun are piggy-back basins. Figure generated with ENVI Classic (https://www.l3harrisgeospatial.com/Software-Technology/ENVI) and Adobe illustrator CS6 (http://www.adobe.com/fr/products/illustrator.html).

In this paper, we present morphological and paleoseismological investigations along this fault in western Nepal. The study area is located between the towns of Surkhet and Gorahi (Figs. 1, 2) where the fault exhibits a clear ~ 120 km-long fault trace with a cumulative scarp of up to ~ 30 m; in this region some first-order morphological studies have already been carried out^10,11,14^). The ridge topography also provides a natural high ground, protecting buildings from landslides. Many villages and tens of schools are therefore distributed along the active fault scarp. Based on field observations, aerial photos, satellite images and published geological maps, we compile and revise geological, morphological and structural data to establish a precise mapping of the fault trace and summarize its morphological characteristics. To evaluate how earthquakes on the Reactivated Boundary Fault relate to major earthquakes on the frontal thrust, we obtain chronological constraints on the last event in an excavated trench. From there, we propose a model for how the hanging wall extension relates to the overall compressive fault system, and discuss implications for the Himalayan thrust system seismic cycle.

## Geological setting

The collision of the Indian plate with the Eurasian plate over the last 50 Ma has formed some of Earth’s most striking topography, including both the Himalayan mountain range and the Tibetan Plateau to the north. Along the Himalaya, this collision is accommodated largely by slip along the Main Himalayan Thrust (MHT) plate interface at ~ 12–21 mm/year (e.g.^20–25^), with a long record of deformation preserved in the hanging wall rocks. From north to south, this sequence of largely forward-breaking thrusts that branch from the MHT are the Main Central Thrust (MCT), the Main Boundary Thrust (MBT) and the Main Frontal Thrust (MFT) (Fig. 1a,b). In the present fault geometry, direct observations of active deformation are limited, as we see only a portion of the seismic cycle, but in general elastic deformation accumulates around the base of the fault system during the interseismic period, and is then released via updip slip as large (M7) and great (M8+) earthquakes rupturing the interseismically coupled parts of the MHT (e.g.^22–25^, Fig. 1b). These slip events sometimes breach the surface along the most frontal fault, the MFT^20–23,26,31–34^, although some events remain blind (e.g. Mw7.8 2015 Gorkha).

Just tens of kilometers north of the hanging wall of the MFT, an impressive north-dipping active fault cuts the land surface. This fault system resembles the landward-dipping normal faults observed in marine accretionary prisms^5^. This active secondary out-of-sequence fault system, the Reactivated Boundary Fault (RBF), is particularly well expressed in western Nepal, but has also been observed in other places, such as further west in India and in eastern Nepal (e.g.^10,15,16^). Some studies^10–14,16^ have observed this understudied but important and highly unusual fault structure, which appears to have experienced significant recent slip at the surface. Stratigraphically, the RBF lies in the immediate vicinity of the Main Boundary Thrust (MBT), which is the boundary where the Precambrian Lesser Himalayan rocks are thrust over the Mio-Pliocene sandstones of the Siwaliks to the south (Fig. 2). The MBT is generally considered to have been the Himalayan frontal thrust during the Miocene (e.g.^35^), and was later abandoned for more frontal thrust systems (Main Dun Thrusts and Main Frontal Thrust). Based on the proximity of the RBF to the MBT, the recent movement on the RBF that we document is likely associated with out-of-sequence reactivation of the Main Boundary Thrust fault system at depth.

**Figure 3:**
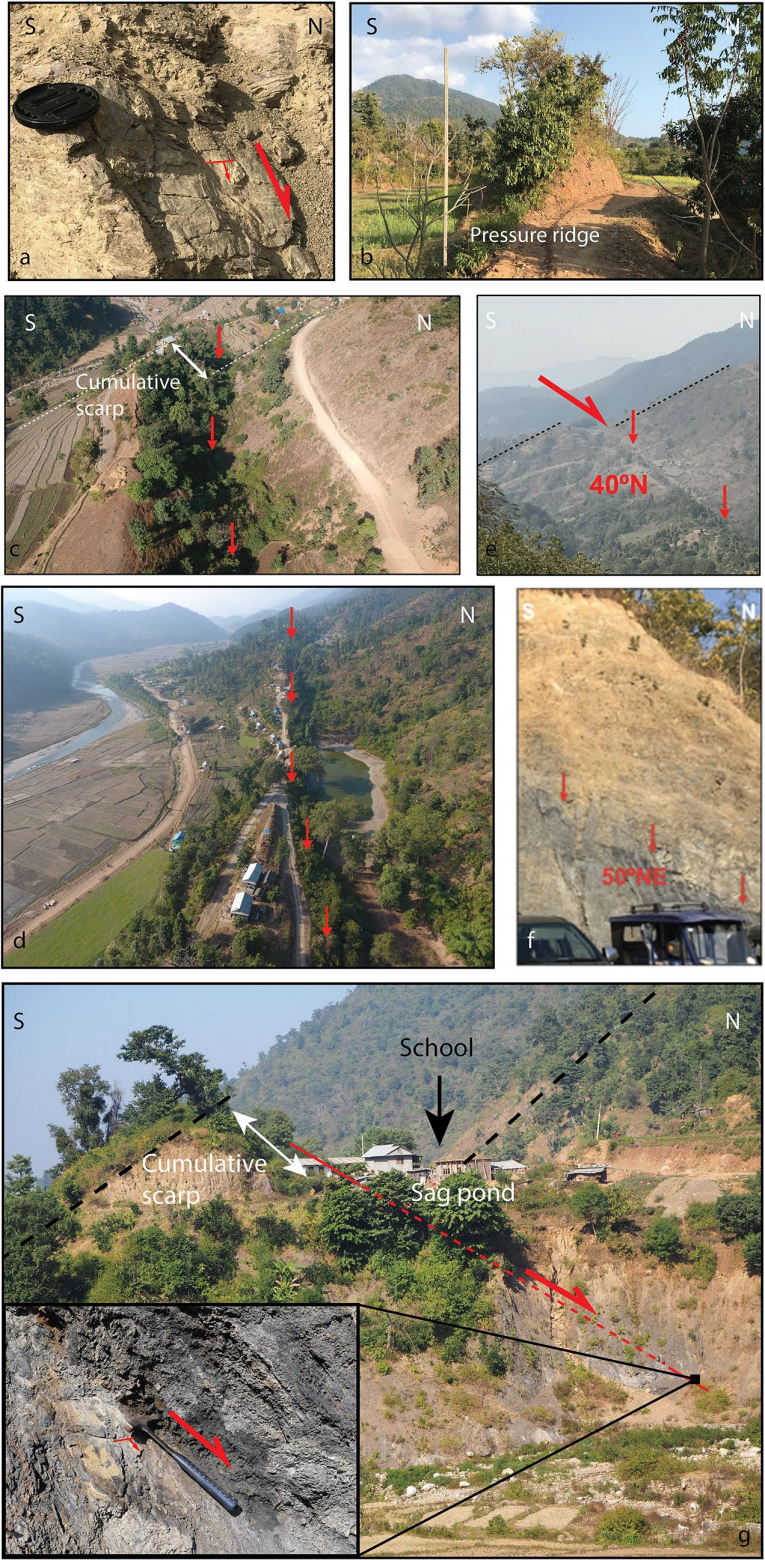
Field pictures along the fault located on Fig. 4a. (**a**) Normal fault centimetric slickensides, (**b**) Topographic expression of the ridge, (**c,d**) cumulative scarp and sag pond within the Bheri-Sarda corridor, (**e,f**) dip of the fault and (**g**) large outcrop across the fault. The fault affects the black slates of the lesser Himalayas. Fault plane affecting the black schists. Kinematic criteria comprise slickensides, with steps normal to the fault surfaces, perpendicular to striae. Slicken lines in the vicinity of the hammer are calcite striae indicating the direction and sense of slip which is consistent with a normal fault (Here the lacking compartment is the hangingwall which collapsed). Figure generated with Adobe illustrator CS6 (http://www.adobe.com/fr/products/illustrator.html).

**Figure 4:**
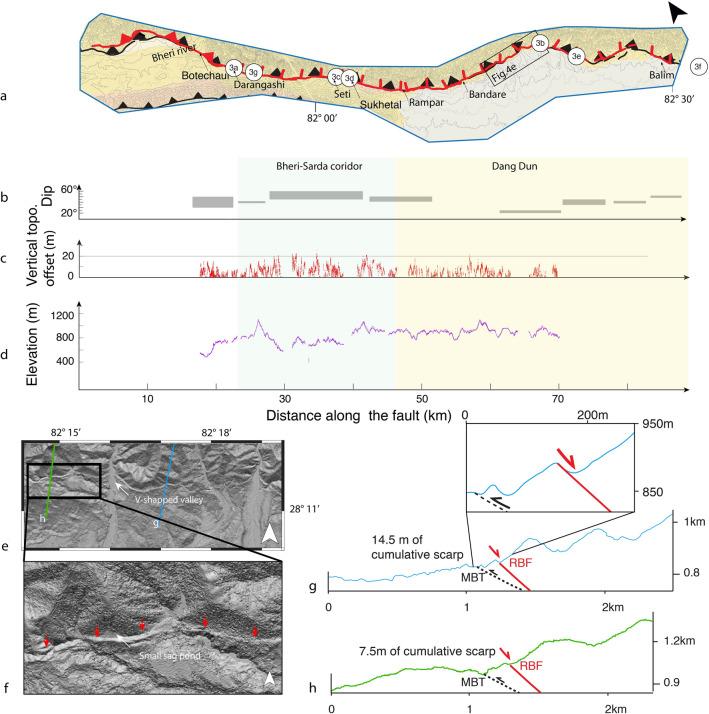
(**a**) Expanded view of the tectonic map of the Surkhet-Gorahi segment of the Reactivated Boundary Fault with location of field pictures from Fig. 3. (**b**) Dips derived from V shaped valley and field measurements on the fault (**c**) Vertical offset of the cumulative scarp topography or ridge height and (**d**) Elevation of the top (red) and bottom (blue) of the cumulative scarp or ridge. (**e,f**) Digital elevation model near the fault 0.5 m resolution. Red arrows locate the fault trace. (**g,f**) Topographic profiles across the fault trace located on (**e**). Digital elevation model near the fault 0.5 m resolution. Figure generated with ENVI Classic (https://www.l3harrisgeospatial.com/Software-Technology/ENVI) and Adobe illustrator CS6 (http://www.adobe.com/fr/products/illustrator.html).

In western Nepal, even though the RBF is well exposed over several hundreds of kilometers with a clear morphological signature (Figs. 1a, 3, 4), the time of the last earthquake on this fault remains poorly constrained. In general, the earthquake record in western Nepal has been less studied than in eastern Nepal, in part due to the long time interval since the last major earthquake (> 500 years). However, according to historical records, earthquakes in the west might have been larger than elsewhere in Nepal, possibly exceeding magnitude M8.5^36,37^. Due to the limited paleoseismic record, an accurate assessment of the seismic hazard would require a better understanding of the return period, slip distribution, and lateral extent of such earthquakes. While historical records from that time are limited, there is documentation of a great earthquake in this region in 1505 CE^38,39^, with a surface rupture described in the Mohana Khola (Fig. 1a^31^), where a paleoseismic trench documented ~ 14–24 m of slip. Lacustrine turbidites triggered by strong ground shaking offer the opportunity to extend and improve this record by including blind ruptures: an 800 year long sedimentary archive from the Rara lake (Fig. 1a^40^) indicates eight possible events within that period, although this record cannot distinguish between blind or surface breaching ruptures, local or regional events, or intermediate to great earthquakes. Unfortunately, the inability to relate these turbidites to specific surface ruptures or even to determine if they are associated with near- or far-field earthquakes, precludes using these results on their own. Local neotectonic studies are therefore required to develop a set of potential paleo-earthquake sources that can be compared to this sedimentary archive and help to identify the fault responsible for the strong ground motion.

In western Nepal, evidence of active faulting within the MHT hanging wall has been recognized since the 1980s (e.g.^10,11^), identified from sets of aerial photographs. The authors called this fault segment the Surkhet-Gorahi fault, after the two cities found near both ends of the fault strand. The kinematics of the fault appear complex, with laterally variable surface expression and direction of vertical displacement^10^. Despite a morphological expression of normal faulting along most of its length, the authors also documented reverse faulting according to geological evidence at several places (see Nakata et al.^11^ for specific locations). Based on a qualitative correlation of terraces where the fault crosses the Bheri river (Botechaur 28.427°, 81.884°), and geological evidence along the fault trace, Nakata et al.^10^ proposed predominantly reverse-slip with a dextral component at this location, interpreting a prominent ridge as a pressure ridge, at the base of which they observed crushed Lesser Himalayan rocks overriding young fluvial sediments. Hossler et al.^14^ revisited this location, and reinterpreted the ridge as a fault scarp associated with reactivation of the Main Boundary Thrust (MBT). By correlating and dating the Bheri river terraces, they suggested that the fault at this location ruptures during major earthquakes, accommodating > 8 m of reverse-sense slip in each event, and that it may have been reactivated during the great 1505 earthquake that ruptured the MFT at the Mohana Khola, ~ 30 km to the south^31^.

However, morphological evidence seems to indicate that, for most of its length (east of Daragashi, Fig. 4a), the fault has a predominantly downthrown morphology on its northern, uphill side^10^, most likely associated with normal fault kinematics. Since Nakata’s work^8,9^, Mugnier et al.^12^ documented the sense of motion along most of the fault with microstructural analysis, demonstrating the recent normal fault activity with slickensides and broken pebbles. Since that time, there has been no attempt to characterize the morphology of the fault, and no paleoseismological trenches have been excavated on this secondary fault in Nepal (secondary fault, considering the MHT as the main/primary fault); as a result, the history and behavior of the fault remain obscure.

## Morphology of the Surkhet-Gorahi fault

To further characterize the Surkhet-Gorahi section of the RBF, we first focus on the specific geological and structural environment of the fault, with an emphasis on how its morphological signature relates to the steeper southern flank of the Lesser Himalaya.

### Digital elevation model generation

In order to develop a contextual understanding of the RBF, we use both existing maps and DEMs and develop new, higher resolution DEMs along the trace of the fault. We first built a large-scale structural and geological map of the Surkhet-Gorahi region (Fig. 2) using published geological maps^19,41^ (the Geological exploration map of Nepal by the Petroleum Exploration Promotion Project of the Department of Mines and Geology, Nepal), slightly modified to fit structural and geological field observations and an SRTM (Shuttle Radar Topography Mission) digital elevation model (DEM). We used this map to identify the trace of the fault, and then refined the elevation model near the trace using Pleiades tri-stereoscopic images, acquired on December 21, 2015. These images have a spatial resolution of 0.7 m, and an estimated vertical precision of 1 m.

We then used the Pleiades images to generate a series of Digital Surface Models (DSMs) through an automatic workflow that performs the data co-registration and the DSM generation, as described in Guérin et al.^42^. The data co-registration is based on tie-point detection as a preprocessing step in order to ensure that the image (and hence the DSM) are precisely located. Tie-point detection is particularly sensitive to steep slopes; in order to accommodate this and ensure accurate tie-points, the detection is performed on the images after orthorectification with the most accurate digital terrain model (DTM) available^43^. Following tie-point registration, DSMs are generated for each date, based on so-called ground space image matching with the open source software MICMAC, which was developed by the French National Geographic Institute (IGN)^44^. This methodology allows an elevation value to be calculated for each point on a grid defined with both planimetric and altimetric steps. The final altitude value is chosen by considering the correlation score and a regularization term^42^. Figure 4 represents the DSM obtained from the Pleiades tristereoscopic images. For this study, the DSM was generated with a 2 m planimetric step and 1 m altimetric step.

**Figure 5:**
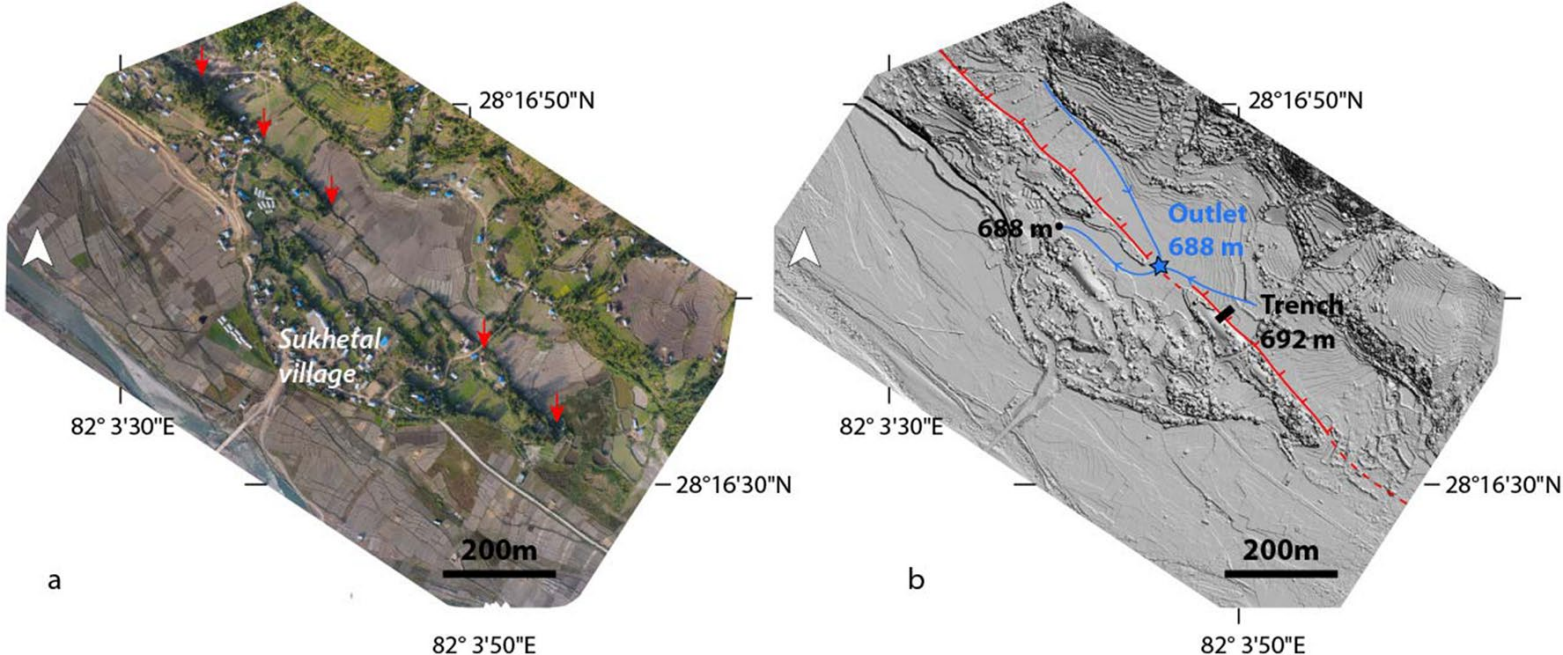
UAV-acquired orthomosaic (**a**) and DEM (**b**) of the Sukhetal area showing the outlet of the river catchment (blue star) at 688 m depth beneath the trench bottom. UAV: Unmanned Aerial Vehicle. Figure generated with ENVI Classic (https://www.l3harrisgeospatial.com/Software-Technology/ENVI) and Adobe illustrator CS6 (http://www.adobe.com/fr/products/illustrator.html).

We generated an additional DSM above our trench site using Unmanned Aerial Vehicle (UAV) photos processed with photogrammetry techniques (27 images, Pix4D, 10 m altitude). This local DSM, with a resolution of 0.1 m, allows us to study the morphology of the river catchment (Fig. 5).

This multi-scale approach allows us to analyze the first-order structures at a regional scale, identify the location of the RBF, and evaluate fine morphological variations along the fault trace.

### Large-scale characteristics and structural position of the RBF in the Surkhet-Gorahi region

On the southern margin of the Himalayan belt, between 81.4° and 82.4° longitude, the frontal fault system is composed of a series of thrusts forming piggy-back intra-Siwaliks basins (referred to as ‘duns’ in the Himalaya) in between low, elongate ridges (the Chure range to the south, up to 1300 m high) (Fig. 2). The orientations of the thrusts and stratigraphic layers composing the frontal system follow the general trend of the belt (here northwest-southeast), with a southward propagation of deformation, and the most active thrust, the MFT, located at the southern front of the belt^19^. Further north, following the same trend, the MBT, which has generally been considered inactive, marks where Lesser Himalayan rocks are thrust on top of the Siwaliks.

The RBF can be tracked in this region as a morphological scarp with a northward dip and a generally downdropped northern side. Where this fault can be identified in Nepal and India, its trace is almost always located less than a few tens to hundreds of meters from the MBT—most of the time on its northern side and 20–40 km north of the MFT. The fault scarp is clearly visible on satellite images and our computed DSM (Figs. 4, 5), located at the base of southern flank of the Mahabharat, a mountain range that reaches up to 3700–4500 m high along the southern margin of the Lesser Himalaya. Like the MBT, the RBF has a northwest-southeast trend (strike approx. N150°).

We mapped the RBF on our Pleiades Digital Surface Model from Surkhet (northwest) to Gorahi (southeast) (see Figs. 4, 5). In its western part, the fault follows a narrow valley between the Mahabharat range to the north and the lower Chure range to the south, hereafter called the Bheri-Sarda corridor, and in its eastern part it follows the northern edge of the Dang dun basin. The Surkhet-Gorahi segment of the RBF seems to terminate in the eastern part of the Dang dun to the east and in the northern part of the Surkhet dun to the west. The trace of the (largely inactive) MBT can be difficult to locate based on morphology, although it represents a prominent lithological contrast. We tentatively map the contact between the Lesser Himalayas and the Siwalik (i.e. the MBT); a comparison between this contact and the RBF shows that the RBF is mostly located on, or slightly north of, the MBT (Figs. 2, 4).

The morphology of the RBF is striking. Along its trace we observe numerous sag ponds, offset terraces and an asymmetric shutter ridge with a steep uphill flank (35°) and gently dipping downhill slope (see Figs. 3, 4). These observations were to first-order described by Nakata et al.^10^, who proposed that the diverse materials composing what they called the “pressure ridge” varying along the trace were due to “the variation in surrounding materials of shattered bedrock filling the pressure ridge.” Near Botechaur, Nakata et al.^10^ describe uplifted terraces north of the fault with a 20 m-high ”pressure ridge” truncating the terrace along the fault trace. Offset terraces were confirmed by Hossler et al.^14^, which reinterpreted the pressure ridge as a fault scarp and dated the terrace abandonments; they interpreted two earthquakes, one occurring after 87–244 CE and the other after 1285–1397 CE.

For most of the length of the RBF in western Nepal (near the Seti, Rampar and Bandare Khola, see Fig. 4), Nakata et al.^10^ document an apparent down-throw on the uphill side of the “pressure ridge”. From Botechaur to 5 km further east, they locally observed shattered Lesser Himalayan rocks spread across a low-angle fault plane on the downhill side of the ridge, apparently thrusting the Lesser Himalaya southward over Quaternary gravel beds, or in some places over Siwaliks, and potentially steepening at depth. They describe the fault as occasionally forming foothills along the steep Lesser Himalayan slopes. Their final interpretation is that there is a contradiction between (1) the topography expressed by the mountain slope, with apparent down-throw of river terraces uphill, and (2) the reverse sense of displacement derived from the geology. They attribute this contradiction to locally limited intrusion of shattered Lesser Himalayan rocks along the fault forming both the superficially low-angle thrust downhill and the apparent down-throw uphill. They also observed dextral displacements of the Seti and Balim Khola rivers.

Our own observations reveal sparse evidence of a plurimetric asymmetric shutter ridge (2 to 30 m-high), with a steeper slope uphill at several places along the fault trace (Fig. 3b, 4e). Folded layers of colluvial or alluvial units composed of fine sediments, gravels, pebbles or clasts are exposed within this ridge, most often at its top, while the core of the ridge largely consists of fragmented Lesser Himalayan rocks. Although the topographic expression of the fault has been degraded by erosion, we observe for most of its length, an apparent downdrop of the northern side associated with a cumulative scarp. Figure 3g shows a ~ 50–70 m-high outcrop where the fault dip (35–40° N), slickensides and cumulative scarp can be observed. On this outcrop, the fault affects the black shales. Kinematic criteria comprise slickensides, with steps normal to the fault surfaces, perpendicular to striae. Our high-resolution Pleiades DSM allows precise mapping of the fault trace in 3D (see Fig. 4). The strike of the fault varies laterally, following the general trend of the geological structures. From west to east, the strike is approximately N135 following the northern side of the Bheri-Sarda corridor, then a general trend at N150 along the northern edge of the Dang Dun basin, with 10 km-long fluctuations (Fig. 4). The shape of the fault trace as it crosses topography together with field measurements allows us to estimate the dip of the fault; this dip varies from 20° to 60°, reaching a maximum in the Bheri-Sarda corridor and a minimum within the Dun valleys (Figs. 3, 4b).

Topographic profiles extracted from the Pleiades DSM across the fault trace show that most of the south-dipping Lesser Himalayan topographic slopes are offset by the fault (Fig. 4g,h), with primarily normal-sense motion on a north-dipping fault. This sense of motion is confirmed by slickensides found at four different locations (Figs. 3, 4). The profiles highlight the cumulative relative uplift on the south side of the fault (see Fig. 4). The height of the cumulative fault scarp varies between 3 and 27 m along the fault trace between Surkhet and Gorahi according to our 3D mapping and field observations (Figs. 3, 4). The distance between the normal fault and the MFT varies along the Surkhet-Gorahi segment from 35 to 40 km, to 20–25 km respectively east and west of Botechaur. The fault is closely linked to the MBT trace (see Fig. 4). Furthermore, the overall surface expression of the fault within the Surkhet-Gorahi segment is well-expressed east of Botechaur, as described above, whereas it is harder to follow between Botechaur and Surkhet. This does not seem related to a lateral variation in erodibility, but to a lesser development of the scarp at the western tip of the reactivated segment. This is likely related to the change in fault dip and sense of offset, both tied to the existence of a scissor fault near the western end of the structure, with reverse sense of motion in Botechaur and westward.

Overall, the offset of the topography, the normal fault slickensides and the development of secondary features such as wet and dry sag ponds along the active fault trace east of Botechaur (from 5 to 60 km eastward), are consistent with recent activity of a north-dipping normal fault, with the downthrown side in most cases to the north (see Figs. 3, 4). Indeed, the particular, upslope-facing scarp morphology of the fault enables the development of small ponds^10^ and endoreic basins during surface rupturing earthquakes, which can then record sedimentation post-earthquake. The development of an asymmetric ridge at some places along the fault together with observations of the Lesser Himalayas overthrusting the Quaternary gravel layers^10^ might indicate the presence of the superficially low-angle thrust even though we did not directly observe this particular contact nor the fault plane.

## Paleoseismic observations at the Sukhetal trench

### Morphological observations of the Sukhetal watershed

In the south-eastern part of the Bheri-Sarda corridor, there are numerous wet and dry sag ponds north of the RBF trace (e.g. Fig. 3d,g). Sukhetal Village in the district of Suketal is located halfway between Surkhet and Gorahi within this corridor. Most of the village is built along the fault trace, with several houses directly on top of the fault scarp, which dominates the landscape. At this location, the fault trace deviates from the main topographic slope of the Lesser Himalaya, forming a small basin. The potential deposition of fine stratigraphic records within this basin makes it an interesting target for our paleoseismic excavation (Fig. 4). Because we were not able to obtain approval to dig at the basin outlet on the fault trace, and because the water was located only 1 m below this point complicating the trenching process, we did not excavate at the outlet but rather ~ 150 m to the east (Fig. 5). Using our Pleiades DSM, we calculate that the Sukhetal watershed drains an area of 0.8 km^2^ with a maximum headwater at 1340 m elevation and steep slopes. The outlet is located at 688 m elevation at the fault trace, i.e. 4 m deeper than at our trench site. The prominent fault trace in the northern part of the village is marked by an ~ 8–10 m high cumulative scarp that continues for ~ 400 m. It is likely that when the fault slips, the scarp dams the small basin and is followed by high post-earthquake aggradation (Fig. 5).

### Description of the stratigraphic units

**Figure 6:**
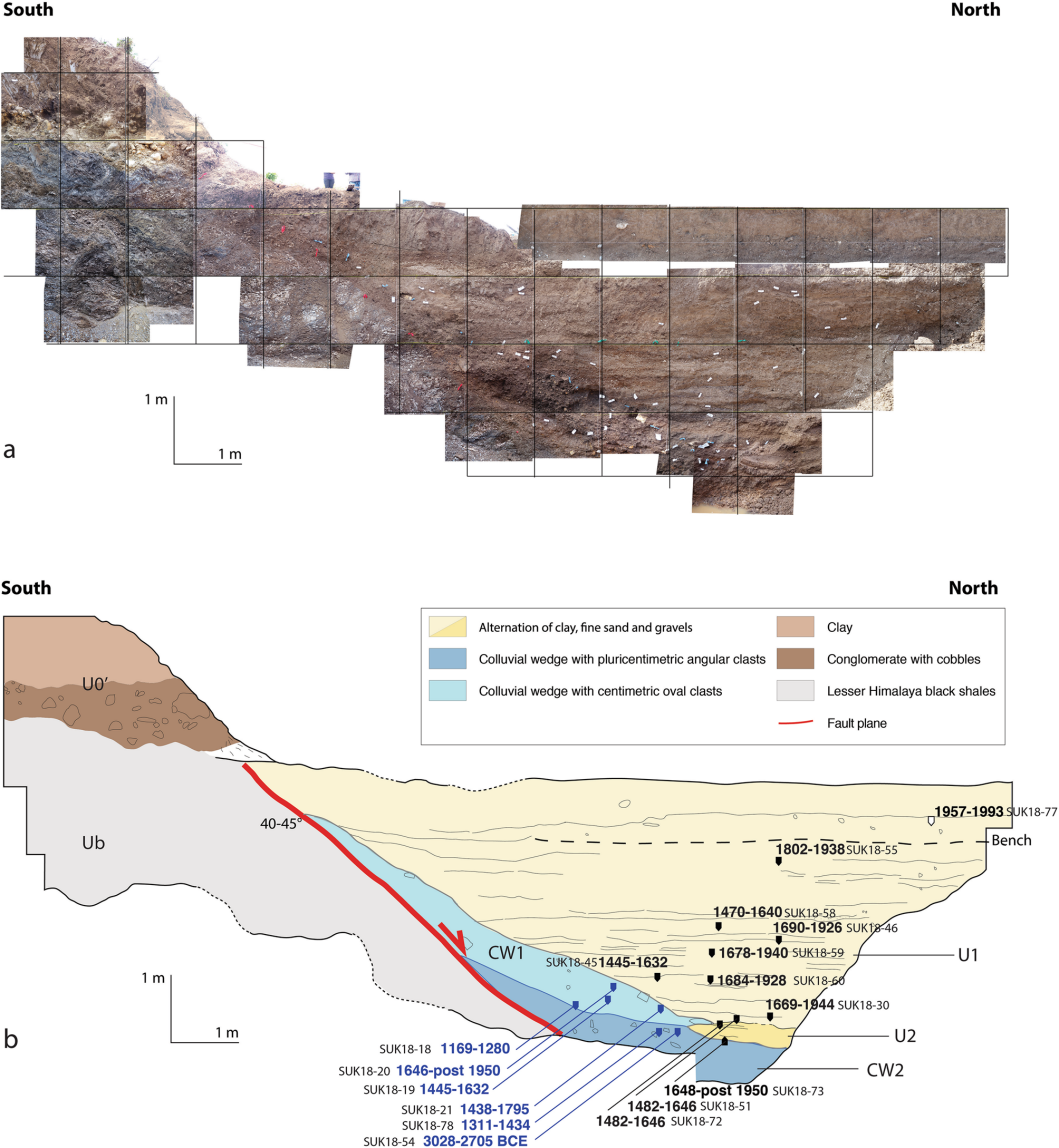
(**a**) Ortho-mosaic of the Sukhetal trench eastern wall. The orthomosaic and log of the trench eastern wall are mirror images of the real observations (flipped N–S) to simplify the comparison to classic Himalayan sections. (**b**) Interpreted log of the trenchwith the location of 18 radiocarbon ages from detrital charcoals (in the colluvial wedges and stratigraphic units in blue and black, respectively). The minimum and maximum at 2 sigma of the individually calibrated detrital charcoal calendar ages are reported. The ages associated to the charcoals found in the colluvial wedges are reported in blue. CW1, colluvial wedge brown-reddish unit of gravels and pebbles, in light blue, CW2, colluvial wedge incorporating pluricentimetric clasts, in dark blue. Units U1 in light yellow, U2 in dark yellow, For sample analysis see Table S1. Figure generated with Adobe illustrator CS6 (http://www.adobe.com/fr/products/illustrator.html).

In order to better understand the slip history on the RBF, we excavated a trench across the fault trace at Sukhetal (82.0635° E/28.2776° N). The trench was 17 m long, 3 m wide and 4.5 m deep, oriented north–south across the scarp with two benches on each side to avoid collapse of the trench walls. Figure 6 documents the trench’s eastern wall. The orthomosaic and log of the trench eastern wall are mirror images of the real observations (flipped N-S) to simplify the comparison to classic Himalayan sections. In the trench, we observed the following:

1. A sharp contact, cutting from the surface to the bottom of the trench is identified between the bedrock (Ub) alluvial and colluvial sediments, and lines up with a rough topographic slope at the surface above the trench. This sharp contact is interpreted as a 6 m-wide fault plane with a dip of 40°–45°.
2. The footwall, where the bedrock formation is composed of deformed lacustrine limestone/shales. On the footwall side, the upper part of the cumulative scarp is smooth. Ub is topped by a 30–100 cm-thick light-colored cobble layers within a fine sediment matrix (﻿U0’﻿) unaffected by the fault located below the topsoil.
3. The hanging wall: here, the uppermost part of U1 is composed of topsoils together with a 50 cm-thick unit of light-brown silt and fine sediments that show no sedimentological organization. The heterogeneity of this unit relates to anthropogenic tilling of the upper layer during agriculture. Below the topsoils, there is a > 1 m thick unit composed of alternating layers of brown gravels (5–20 cm thick), fine sands (40–70 cm thick) and dark clayey horizons (< 3 cm thick), the latter being more frequent towards the bottom. U1 is deposited above a wedge of unsorted colluvial material, 1 m-thick and 6 m-long, thinning northward (Colluvial Wedge 1: CW1), containing yellow and greyish clasts within a dark-brown clayey and sandy matrix. CW1 is deposited above U2 (contact only evident at the northern tip of CW1), which exposes alternating brown-orange sandy horizons (< 10 m-thick) and brownish clays (20–40 m-thick), with a few black pockets. U2 is deposited above CW2, a massive colluvial wedge unit containing large pebble and cobble-sized clasts of angular limestones/marls together with granular yellow to orange cobble-sized material within a brownish matrix. Near the fault, CW1 lies directly above CW2 with no intervening U2; the relationship with U2 is only visible on the northern side of the trench. The base of the trench is limited by the groundwater level, which lies at 4.5 m below the surface. The two units of debris (CW1 and CW2) are undeformed and were deposited on top of the bedrock (Ub). The fractured nature of the bedrock made it difficult to find slickensides within the trench, but normal fault slickensides were found at the same structural position a few kilometers west and east of the trench (Fig. 3 for pictures and 4 for locations). A steeper dip of 60°–65° was found for the fault plane in a pit dug 5 m east of the trench, indicating significant local fluctuation in dip along the fault, at least near the surface.

### Radiocarbon dating of detrital charcoal samples from the trench

We collected 80 charcoal samples from the trench and dated 18 of these to constrain the timing of deposition of the principal units. The charcoal samples were prepared and dated by Beta Analytics, Miami, Florida. Thirteen samples were dated by AMS (Accelerator Mass Spectrometry); four were too small for AMS and were dated by Micro-AMS instead (see Table S1). The samples were individually calibrated using radiocarbon calibration Oxcal V4.3 and the IntCal13 atmospheric calibration curve^45^. For sample SUK18-77, which is considered as modern (post 1950), we used the Bomb04NH1 calibration curve^46^. Radiocarbon ages range from 3028 BCE to 1993 CE.

Combining the ages with observations from the trench, we interpret that colluvial wedge CW2 (4 samples: SUK18-54, -78, -18, -53) was deposited before U2 (3 samples: SUK18-73, -72, -51). The most recent ^14^C calibrated age within CW2 is younger than 1311 CE, indicating that the wedge was necessarily deposited after 1311 CE. U2 and CW2 are undeformed, implying that no local slip has occurred on the fault since the deposition of CW2, which could have happen at any time after 1311 CE. The ages of the three charcoals collected in CW1 (SUK18-21, -19, -20), together with the ^14^C calibrated ages from U1 (8 samples: SUK18-30, -60, -45, -59, -46, -58, -55, -77), imply that it was necessarily deposited after 1646 CE. Based on the individually calibrated ages, the undisturbed 4 m-thick fluvial sediments composing U1 and U2 suggest a minimum sediment accumulation rate of 15 mm/year at least since the seventeenth century. The obtained ^14^C calibrated ages do not account for the inbuilt age (age of the wood at the time of burning) or the time of transport before deposition. Therefore, the ages associated with the collapse wedges are maxima (older than the deposition age), and the deposition rates minima.

**Figure 7:**
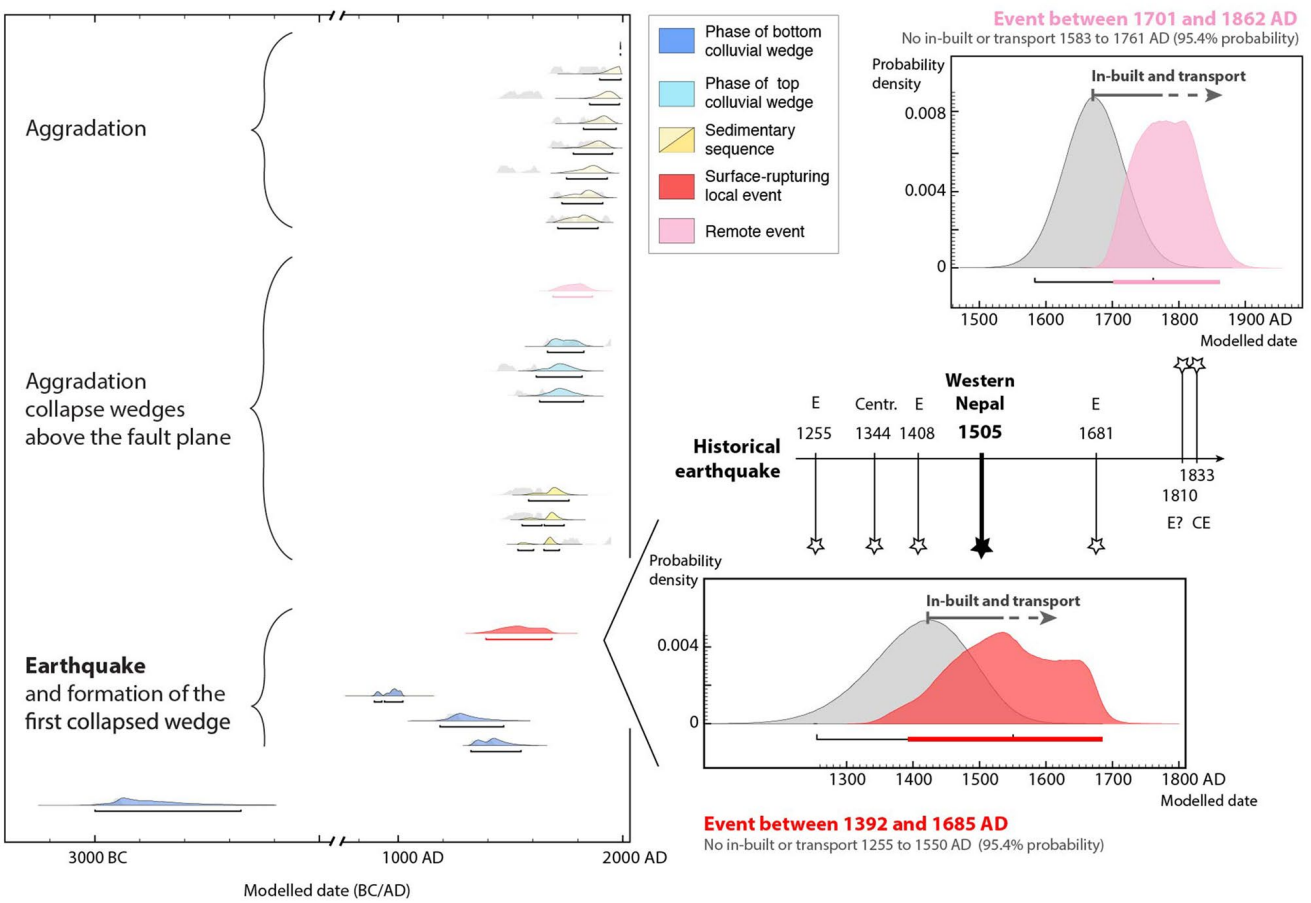
Detrital charcoal radiocarbon ages with inbuilt age as a function of the depth. In gray, calculated probability density spectra for possible ground shaking events on -or off- fault. The maximum and minimum sedimentation rate are represented in blue. Figure generated with Adobe illustrator CS6 (http://www.adobe.com/fr/products/illustrator.html).

To refine the chronology of events, we added stratigraphic information by combining phases (unit with no defined stratigraphic relations) and sequences (unit with a defined stratigraphic order) using the Oxcal software^47^. We tested several models combining these sequences and phases, also including the charcoal outlier model to account for the inbuilt age offset, age-to-death time, and “old wood effect,” in order to build a more refined model^48^. The charcoal time of residence on the site is likely 10–100 years^48^. A recent study on the precision of Bayesian modeling for samples susceptible to inbuilt age revealed that the Oxcal charcoal outlier model, which allows the addition of constraints on the average age of the trees, can be used to correct the ages most accurately, with their 95% probability range encompassing the known true date for all tested samples, resulting in a robust chronological model^49,50^. Probability density functions modelled with and without inbuilt age and transport for the events associated with CW1 and CW2 are shown in Fig. 7 in color and in grey, respectively. Because the trees in the area are not considered to be long-lived species (based on personal communications with the villagers on the age of the oldest trees), we set the inbuilt offset scale to a maximum of 100 years instead of the usual 1000 years^48,50,51^ to obtain a more accurate older boundary for the age of deposition. We fixed the age of the uppermost sample (which is the only modern sample) at 1990–1991 CE based on its individually calibrated age; this sets the youngest boundary of our model. Units U1 and U2 are considered to be stratigraphically organized with depth (set as sequences), while the colluvial wedges CW1 and CW2 are classified as unordered phases. In this more realistic model, the trench sequence is as follows: CW2, U2, CW1 and U1. The 95.4% PDFs (probability density functions) for the events associated with the collapse of wedges CW1 and CW2 respectively range from 1690 to 1865 CE, with the highest probability around 1800 CE, and from 1393 to 1684 CE, with the highest probability around 1500 CE (see Fig. 7). A larger charcoal inbuilt age and an additional phase of transportation shifts the deposition ages toward more recent dates by a few decades.

## Discussion: Recent activity of the Reactivated Boundary Fault and implications for the mechanics of the Himalayan frontal fault system

The combination of the new geomorphological and chronological data with historical observations can be used to constrain the recent slip history of the fault, giving insights into the dates of paleo-earthquakes, the local sediment accumulation rate and the normal fault position within the overall compressional Himalayan thrust system.

### Uncertainties of the data and limitations of the methodology

The epistemic uncertainties on the recent kinematics along the fault remain elevated due to polyphase deformation, the tectonic expression of the fault, and the limited sites at which we see fault mirrors including fault kinematic indicators.

The association of historical earthquakes to the paleoseismic record excavated in the trench remains challenging due to several limitations. First of all, the calibrated radiocarbon ages (^14^C-derived ages) are associated with variable uncertainties, sometimes larger than a century when the slope of the calibration curve is flat. In this study, these uncertainties are first individually determined using the Reiner et al. intcal13 calibration curve^45^. The uncertainties vary significantly over the epoch covered by the trench wall’s samples, from (1) a few months for post-bomb samples, to (2) a few decades for medieval samples and (3) larger values, exceeding a century, for wood samples that grew during the 18th to early twentieth centuries. Second, the samples dated being detrital charcoals, the ^14^C-derived ages do not allow direct dating of the deposition time of the sedimentary unit, but predate it by the age of the wood at the time of deposition. This time span depends on the inbuilt age of the sample (age of the wood at the time of its death) and the duration of transport before deposition. As recognized by Bollinger et al.^52^, this duration in Nepal is locally as small as a few decades. However, these observations cannot definitively exclude larger values, where long-lived tree species or long residence time processes dominate.

One way to simplify the dating of the events described in the trench is to take the youngest charcoal from every unit and constrain a “termini post quo” model based on individually calibrated ^14^C-derived ages, which necessarily predate the event by a time span greater or equal to zero years. We refined this model, following a Bayesian approach^45,49,50^. We tested models with and without inbuilt and transport ages. We tested models taking into account an inbuilt age distribution of 10–100 years, values that appear consistent with the present-day intermediate-lived tree species and the distribution of detrital ages. We also tested less likely models with larger values (inbuilt age distribution at 1000 year), and find that they do not change our conclusions.

Another limitation comes from the fact that our trench is only 4 m deep due to the presence of the water table and the unavailability of a water pump for dewatering. This prevented us from determining the amount of coseismic slip on the normal fault plane. Thus, our estimate of the displacement on the fault is tentative and should be taken as a minimum displacement.

Despite these limitations and significant uncertainties, the results and timespan encompassing the possible dates for the last event are robust and can be compared to the time series of significant earthquakes that have been independently documented in western Nepal during the time frame covered by the sedimentary record.

### Implications of the paleoseismology survey, past events and historical observations

**Figure 8:**
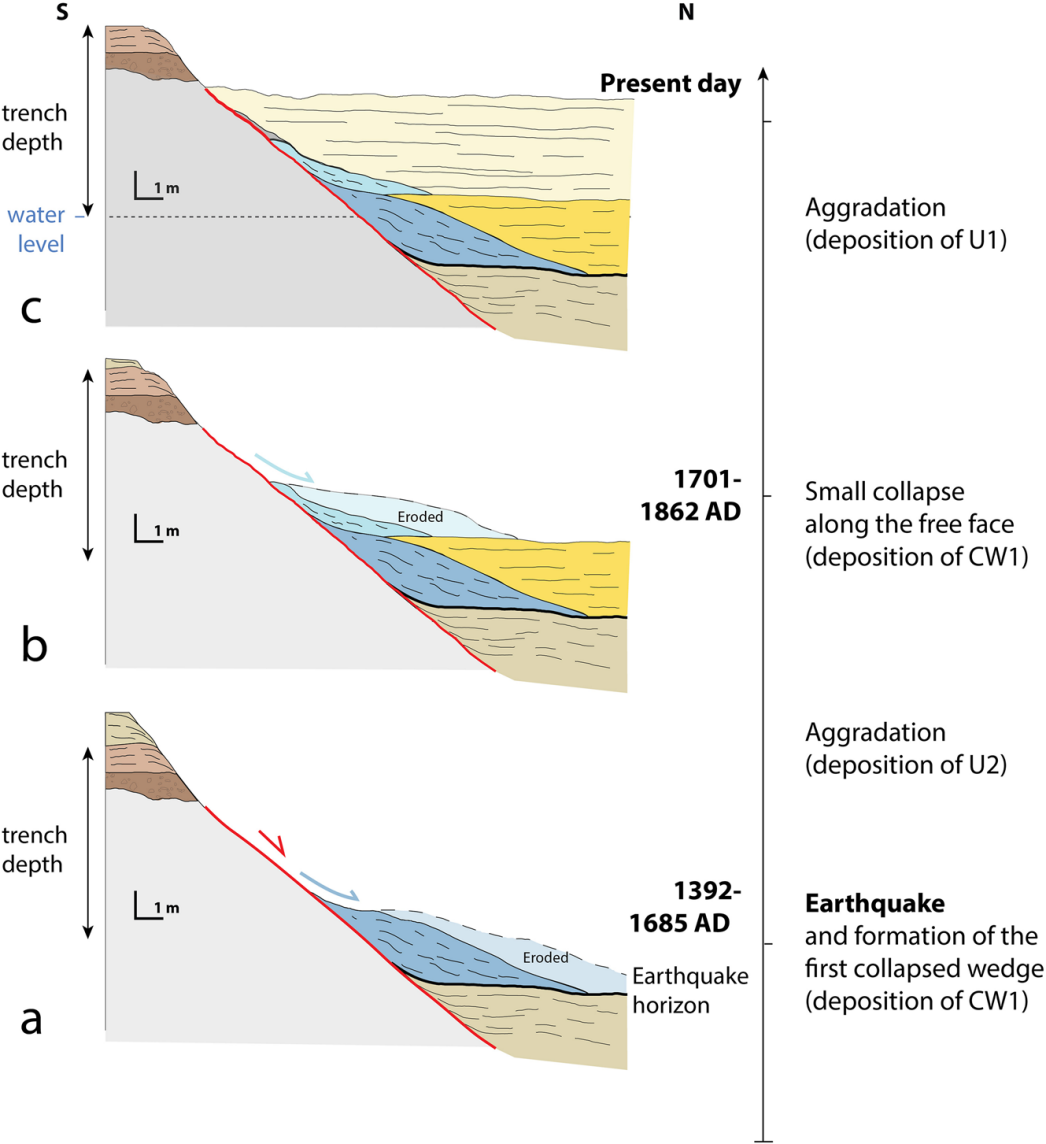
Schematic reconstruction of the Sukhetal site. The details concerning the lithological nature of the units and their colour code are shown Fig. 6. Figure generated with Adobe illustrator CS6 (http://www.adobe.com/fr/products/illustrator.html).

The Sukhetal trench yields information about the deposition of two colluvial wedges at the toe of the RBF scarp, which must be related to ground shaking events, climatic events or dismantlement of the scarp (e.g.^34^). Our proposed interpretation for the recent history of the Sukhetal river catchment aggradation and the seismic events is shown in Fig. 8. The collapse of colluvial wedge CW2 is associated with a surface-rupturing earthquake (EQ) occurring before 1684 CE and most probably after 1394 CE (individually calibrated radiocarbon age at 1311–1434 CE in CW1). The probability density function for the age computed with the Oxcal charcoal outlier model indicates a high probability around 1500 CE (Fig. 7).

We considered all historical earthquakes and information on groundshaking reported within this period in historical earthquake catalogues^36,37^. Within western Nepal during this period, the only historical earthquake recorded in the chronicles occurred in 1505 CE^38^. This earthquake ruptured the MFT at the Mohana Khola^31^ and possibly also in Koilabas^41,52^. The fact that collapse and deposition of CW2 at the RBF overlaps with this period makes it likely that this collapse was associated with the 1505 CE earthquake or its aftershocks, due to (1) surface rupture on the RBF during or soon after the 1505 CE earthquake, which produced earthquake-triggered turbitites in the Rara lake records ~ 90 km north of the RBF^40^, and/or (2) the wide-spread groundshaking associated with rupture of the MHT/MFT system in 1505 CE.

Since groundshaking certainly must have occurred in 1505 CE, we cannot discard option (2). However, we note that, compared to the overall rough exposed cumulative scarp along the fault, the contact between the bedrock and the overriding sediments is very sharp. The contact presents no erosion levels along its deeper part, which suggests that there was indeed slip on the RBF itself, causing the formation of an endorheic basin north of the fault scarp and an important aggradation period until the seismic scarp was covered or breached. The linear age-depth profile yielded by our Bayesian model for U1 and U2 suggests near-continuous aggradation from at least 1691 CE, with a minimum sedimentation rate of ~ 8 mm/year and an average value of ~ 15 mm/year (Supplementary Fig. S1).

The topmost colluvial wedge (CW1) was deposited during this period of aggradation. This wedge cannot be associated with a local earthquake breaching the surface on the fault because the colluvial wedge overlies units CW2 and U2, which appear undeformed. Thus, this collapse was due to either groundshaking associated with a significant regional earthquake, or some non-tectonic process, such as erosion or anthropogenic modification. The chronological constraints and the Oxcal Bayesian model suggest that this event (E) occurred between 1690 and 1865 CE, with a maximum probability in the early nineteenth century. Within this period, only two historical earthquakes have been documented in chronicles: in 1803 and 1833^53–55^. The 1803 Kumaon and 1833 Central Nepal earthquakes have been recognized as being large events (M7+) with the capacity to eventually generate intensity IV–V shaking at the site (exposed to intensities around V following the intensity attenuation law of Szeliga et al.^56^). This event (E) could also potentially postdate the period 1690–1865 CE by a few decades, provided the charcoals are associated with a transport time lag and/or larger significant inbuilt age. Two large earthquakes happened later in the twentieth century, the M8.4 1934 CE Eastern-Nepal earthquake, which would have been associated with intensity IV-V shaking at the longitude of the site^55^. Only the 1936 CE M6.9 earthquake, with an epicenter 125 ± 30 km away (ISC-GEM catalogue), was probably associated with intensity VI shaking (following Szeliga et al.^56^ attenuation law); this strong ground shaking could have caused seismic collapse. Evidence for strong ground shaking in the region has also been highlighted by eight turbiditic events between 1135 and 1947 CE in the Rara lake sediments^40^. However, there is insufficient evidence to correlate a specific groundshaking event with this collapse, or to infer that it was tectonic as opposed to climatic or anthropogenic.

### Implications of the morphological survey

Using the Pleiades DSM, we map out the 3D shape of the fault trace, allowing for a more precise localization. To first order, the resulting trace is consistent with the location derived from aerial photographs, topographic maps and field observations by Nakata et al.^10^. However, our more detailed morphological and paleoseismic observations suggest normal fault motion within the Bheri-Sarda corridor and the Dang dun basin, which is consistent with the interpretation of Mugnier et al.^12^ but opposed to the reverse-dextral motion proposed by Nakata et al.^10^ based on geological observations. Dip-slip slickensides with normal-sense movement found at three locations along the fault also indicate that the recent surface-rupturing event did not accommodate dextral motion. Along most of the fault trace, a clear normal offset in the topography of the Lesser Himalayas is visible. However, at several locations along the fault, we observe ~ 3–10 m high and ~ 2–5 m wide ridges composed of folded layers of boulders and fine sediments at their tops and crushed Lesser Himalayan rocks in their cores.

The topographic trace of the fault (i.e. ridge or scarp) is well expressed between Botechaur and Gorahi. The observations are sparser and less convincing to the west, between Surkhet and Botechaur. The variations in geomorphological expressions of the active fault observed along strike could be related to differential erosion, geometry and kinematics of the active fault system. Overall, except for first order variations in dip and cumulative scarp between the basins and Bheri-Sarda corridor, the apparent segmentation of the topographic scarp appears to be primarily controlled by the major rivers responsible for erosion of the cumulative scarp.

The throw profile does not depict a typical pattern of multi-segmented fault coalescing (e.g.^57^), which is probably due to the fact that the fault experienced a long and complex poly-phased deformation history.

### Constraints on reactivated boundary fault slip events and implications for great Himalayan earthquakes

To further constrain the RBF slip events characteristics, we speculate about the size of the events that have occurred on this secondary fault, considering the MHT as the main/primary fault. As described above, the linear age-depth profile yielded by our Bayesian model for U1 and U2 is consistent with an average sedimentation rate of ~ 15 mm/year since 1691 CE (Supplementary Fig. S1). As a coarse estimate, we consider the possibility that this sedimentation rate has been near-constant since the last earthquake, such that slip on the fault created a local depocenter. The downward extrapolation of the sedimentation rate of ~ 15 mm/year at depth suggests that the probable age of the colluvial wedge (from the 1505 CE earthquake or its aftershocks) would be reached at ~ 6 m below the surface (2 m below the trench bottom, i.e. water level), possibly indicating (1) the depth at which the post-event aggradation started and (2) the base of the basal colluvial wedge (CW2).

The contact between the bedrock and the overlying recent sedimentary units is 9 m-long total, due to successive displacements. Six meters of this length shows an absence of erosion along this sharp contact, suggests that the surface formed as a 6 m-long freeface, later covered by a colluvial wedge and a sequence of later sediments. Considering the high erosion potential in this area and the absence of a paleosoil or erosion notch, the sharpness of the observed fault trace advocates for a single event.

The deeper colluvial wedge (CW2) is not deformed and may be associated with the last surface-rupturing slip event on this fault. The sharp ex-freeface implies a minimum of 6 m of slip for this last event on the 9 m exposed fault plane, to which the buried thickness of CW2 must be added (probably 2 m based on the extrapolation at depth of the minimum sedimentation rate). Relying on this hypothesis, a magnitude of Mw7.2–7.4 can be inferred from empirical laws for slips between 6 and 8 m on typical normal faults^58^. However, based on our observations, the RBF seismic behavior might not be typical, as normal events on the RBF could be triggered by great earthquakes on the MFT; in addition, the RBF terminates at depth into the MHT, which generally slips in the opposite (thrust) direction. In this case, it is possible to estimate the event parameters as a function of the seismic moment released. Hanks and Kanamori^59^ moment magnitude scale indicates that a Mw 7.2–7.4 earthquake with 6 m of slip could be produced by a 120 km-long, 6–7 km wide rupture, consistent with the extent of the fault trace observed for the Surkhet-Gorahi portion of the Reactivated Boundary Fault. The minimum 6 m-long ex-freeface and the colluvial wedge CW2 may therefore be associated with a large (Mw7+) surface-rupturing earthquake on the fault, plausibly associated with the great 1505 earthquake or its aftershocks that ruptured the MFT and produced groundshaking across a wide area.

As mentioned previously, lateral variations of fault expression are significant along the range: ten to hundred kilometers-long active segments were mapped along strike, sometimes interrupted by long sections where the fault activity does not outcrop. The RBF is also observed in India, ~ 50 km west of Nepal. Philip et al.^16^ describe a fault segment (the “Logar Fault”) that cuts across an alluvial fan, dropping the hanging wall to the north (Fig. 1). This fault segment is only ~ 10 km long, but clearly demonstrates Holocene normal motion, with inferred footwall uplift rates of 2–3 mm/year, based on trenching^16^. Based on their findings, the youngest date of activity of the RBF at this site is old, 11 and 8.7 ka^16^ suggesting that local segments might be reactivated asynchronously. In eastern Nepal, the normal fault can also be followed for ~ 100 km between 86.24° and 86.74° longiude, indicating that the mechanism causing extension near the MBT is not limited to western Nepal and India (Fig. 1).

Up to now, no relation between the frontal thrust and the fault has been demonstrated; our trench suggests that such a link may exist in western Nepal. This observation is consistent with the terrace abandonment in Botechaur^14^. To complicate matters, although the RBF exhibits predominantly normal motion, the along-strike alternation of reverse (Botechaur Nepal^14^, Darjeeling, India^13^) and normal offsets (most of western and eastern Nepal, this study and India^16^) suggests that the deformation accommodated above the locked MHT décollement is complex. The lateral variation in topographic expression, sense of motion and the date of activity of the fault is important, but the mechanisms controlling it are not yet resolved. Several hypotheses can be proposed: triggering of hanging wall extension might happen for exceptional events only (1) after several seismic cycles, or (2) in particular geometrical settings focusing the extension. However, more systematic analyses on secondary faults in compressional contexts need to be done to better understand these mechanisms.

The short distance between the active fault trace of the RBF and the MBT suggests that the development of this extensional structure is strongly controlled by the pre-existing thrust fault geometry. The MBT branches at depth from the Main Himalayan Thrust, the basal décollement of the thrust wedge. Likely the variation in map trace of the RBF compared to the MBT only represents a small deviation near the surface; we suggest that the faults are co-located at depth. The activation of this fault in normal-sense motion indicates that it must have relatively low effective friction, probably because the MBT represents both a major lithological boundary and an old slip surface. The deeper section of the fault developed as a thrust but may have been locally steepened by imbrication by faults to the south, allowing it to reorient into stresses that are more optimal for normal sense slip.

However, it remains a challenge to understand why there is extension occurring in the hanging wall of a major thrust. We suggest several possible mechanisms that can explain this behavior:

1. The wedge, or some part thereof, may have reached over-critical conditions, as proposed by Mugnier et al.^12^. This could be associated with the steepness of the frontal part of the Mahabharat range, fluid pressure variations, or the presence of local ramps on the underlying fault eventually controlled by the geometry of the outer wedge and distance to the MFT, trailing edge of the fault system.
2. The fact that the most coherent strand of the RBF lies in western Nepal may be associated with transtension of the range. The Western Nepal Fault System (e.g.^11,17^), a mapped dextral strike-slip fault system that cuts across the Himalaya, bounds the eastern margin of the fault segment. Such a fault system could produce extension on its western side and compression to the east.
3. Normal-sense slip may occur only during or shortly after large earthquakes on the underlying décollement. Indeed, the largest shallow megathrust events are most likely followed by locally significant stress tensor rotations comparable to those described after the greatest subduction zone earthquakes that could happen during the post-seismic relaxation (e.g. Sumatra 2004 M9,2, Maule 2010 M8,8, Tohoku 2011 M9,0 ^7,8^). Indeed, these subduction earthquakes were followed by local and temporary rotations of the principal stress directions, sometimes exceeding 45°, during a transient period associated with the earthquakes on the normal fault in the hanging wall of the megathrust system^7,8^. If this is the case, then normal motion during some time periods could be reversed during other time periods, potentially explaining both the variability in slip sense, as well as how the scarp records recent movement, with low total cumulative offset. Notably, the most obvious strand of the RBF is in western Nepal, and across the border in India. This is co-located with an area that is suggested to have hosted one of the largest thrust-mechanism earthquakes in the last millennium in the Himalaya, which would be consistent with extension associated with large thrust events.

**Figure 9:**
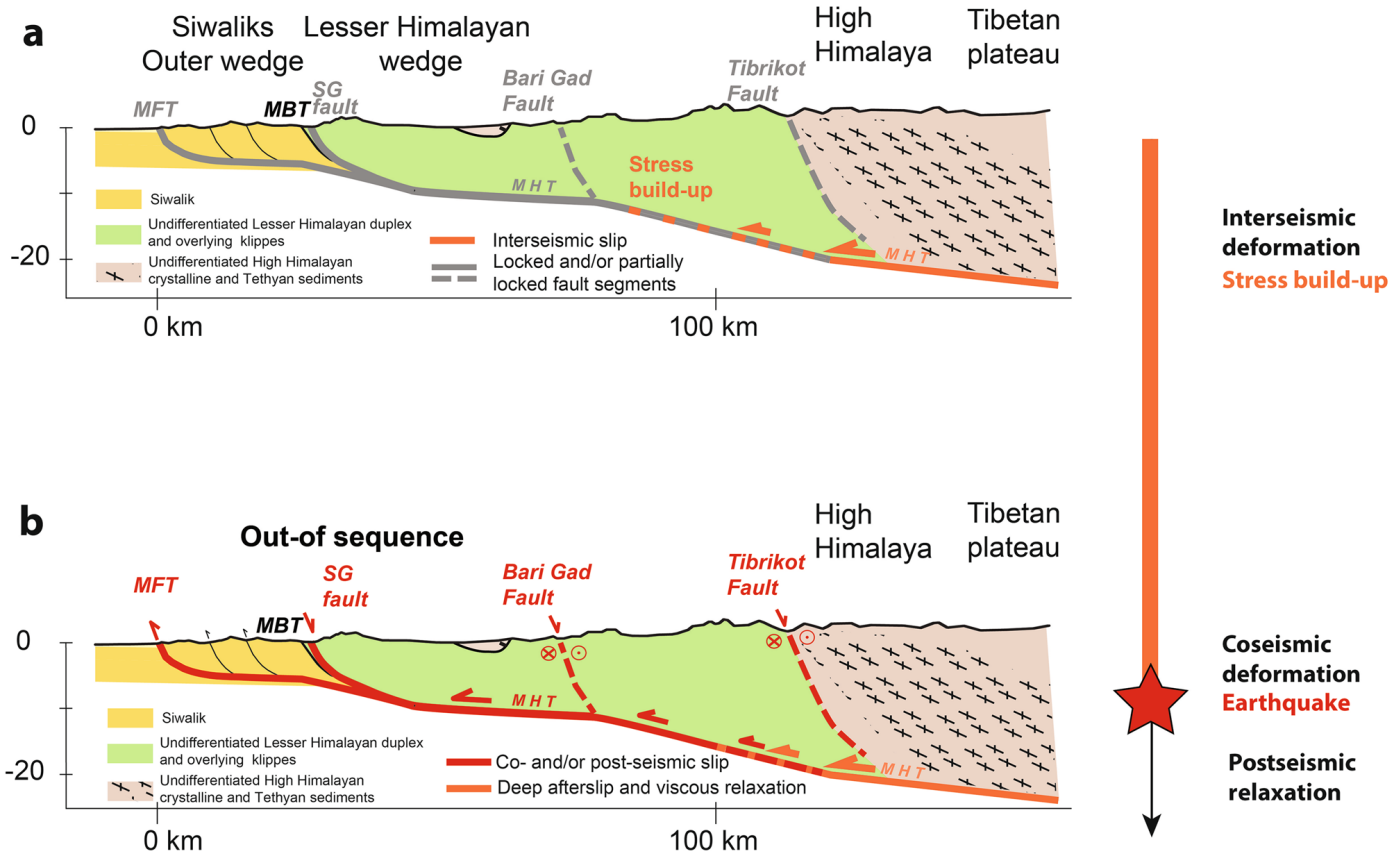
East–West tectonic sketches of the Main Himalayan fault system (see Fig. 1 for location). Figure generated with Adobe illustrator CS6 (http://www.adobe.com/fr/products/illustrator.html).

Our results, as well as those of Hossler et al.^14^, suggest a possible seismic relationship at large scale between the MFT and RBF. The predominantly normal-sense slip on this fault, occurring within a short period of time of a major megathrust earthquake rupturing the front (either co- or slightly post -seismic), suggests that local extension may be a seismic cycle response within a compressional system. This highlights the need to consider the dynamics of the entire wedge to understand the mechanics of the active fault system (Fig. 9).

Considering the present tectonic activity, we hypothesize that the slip motion variations along the secondary ruptures on the RBF could be controlled by several factors, including (1) local variations of the basal friction and rheological conditions on the décollement beneath the trailing edge of the wedge (e.g.^60,61^), or (2) the presence of a ramp beneath the MBT^62^. These conditions may eventually lead to lateral variations in the dynamic response of the wedge, leading for example to local overshooting^63^.

### Implications for the mechanism of megathrust earthquakes and seismic hazard

Based on its position, orientation, and apparent sense of slip, the RBF may be an analog to active faults observed at the front of accretionary wedges overlying tsunamigenic megathrusts. For instance, a moderately dipping (45°) landward-dipping normal fault is visible in seismic data ~ 40 km from the Japan Trench in the hanging wall, and slipped during or soon after the Mw 9.0 2011 Tohoku-Oki earthquake^5^. Numerous normal fault aftershocks have been detected in the hanging wall of the megathrust during the Tohoku crisis, up to a Mw 7.6 event on March 9th^8^. Measurement of the offsets revealed that the offset observed on this fault has been accumulated over multiple earthquakes^5^, as proposed here for the Reactivated Boundary Fault. Seismic reflection images from Japan show that this normal fault branches at depth from the plate interface. The first-order scale of the normal fault in Japan is comparable to the one we describe for the Himalayan frontal system. Similar landward-dipping normal faults have been imaged in other subduction zones using seismic reflection profiles acquired along the Costa Rica-Nicaragua (~ 20 to ~ 50 km from the trench^64^), Kuril (~ 60 km from the trench^65^) and Aleutians (~ 75 km from the trench^9^) convergent margins. McKenzie and Jackson^66^ showed that aftershocks in several convergent margins often have normal fault mechanisms, suggesting that this behavior is ubiquitous. This fault system in Nepal enables us to observe the relationship between thrust earthquakes and normal slip on secondary faults using trenching, a tool not available in subduction zones.

## Conclusion

We document the morphology of a ~ 120 km-long reliefward-dipping segment of the newly named RBF (Reactivated Boundary Fault), an active normal fault ~ 20–40 km north of the Main Frontal Thrust that is often, but not always, coincident with the MBT. In western Nepal, the fault is well exposed, with a cumulative scarp 3 to 27 m high. The topography of the fault and slickensides on the fault plane indicate the RBF exhibits primarily normal sense slip. Excavation of its fault scarp reveals a sharp tectonic contact, overlain by a 4 m-thick alluvial and colluvial pile consistent with filling at a high sedimentation rate of a small endorheic basin created by the upslope-facing scarp morphology of the fault. Radiocarbon ages of these sediments indicate that the aggradation happened after the seventeenth century and demonstrate that the last surface-rupturing earthquake on the fault occurred prior to that period. Joint analysis of the ages determined using charcoals from the basal colluvial wedge and the overlying sediments suggest that the last earthquake rupturing the fault between 1400 and 1700 CE. Furthermore, the location of the RBF fault trace away from the frontal deformation and the interseismic loading zone, and its connection to the MHT at depth together with its normal sense of motion in a collisional context suggest that RBF slip events are triggered by large earthquakes on the MFT. We suggest that the last earthquake that ruptured the Surkhet-Gorahi segment of the RBF occurred during or soon after the latest mega-earthquake on the nearby plate boundary fault, which happened in 1505 CE.

The upslope-facing scarp along the active RBF trace is responsible for the development of wet and dry sag ponds, providing flat areas for agricultural fields and construction. The ridge topography also provides a natural high ground protecting buildings from landslides. Many villages and tens of schools are therefore distributed along the scarp. This active secondary structure and its associated significant seismic potential demonstrate that the fault hazard is important in a larger area of Nepal than previously anticipated. A precise evaluation of the seismic potential of this fault is therefore required to better assess the seismic risks.

The tectonic context of the RBF in the region is of broad interest, being a rare opportunity to document joint ruptures on reverse and normal faults within the hanging wall of a megathrust system responsible for devastating earthquakes (usually described in subduction zones and marine settings).

## Supplementary Information

Supplementary Information.
