# Supplementary material for: Localized extension in megathrust hanging wall following great earthquakes in western Nepal
Source: Sci Rep. 2021 Nov 2;11:21521. doi: 10.1038/s41598-021-00297-4 (PMC8563945; doi:10.1038/s41598-021-00297-4)
Supplement: Supplementary file 1 — Supplementary Information. [file 41598_2021_297_MOESM1_ESM.pdf]

Supplementary material for the manuscript “Localized extension in megathrust hanging wall following great earthquakes in western Nepal”

Magali Riesner (1,2,*), Laurent Bollinger (2), Judith Hubbard (1), Cyrielle Guérin (2), Marthe Lefèvre (3), Amaury Vallage (2), Chanda Basnet Shah (4), Thakur Prasad Kandel (4), Samuel Haines (1) and Soma Nath Sapkota (4)

(1) Earth Observatory of Singapore, NTU, Singapore, Singapore
(2) CEA, DAM, DIF, F-91297 Arpajon, France,
(3) Institut de Physique du Globe de Paris, Paris, France,
(4) Department of mines and geology, Nepal,
*(magali.riesner@gmail.com),

Figure S1

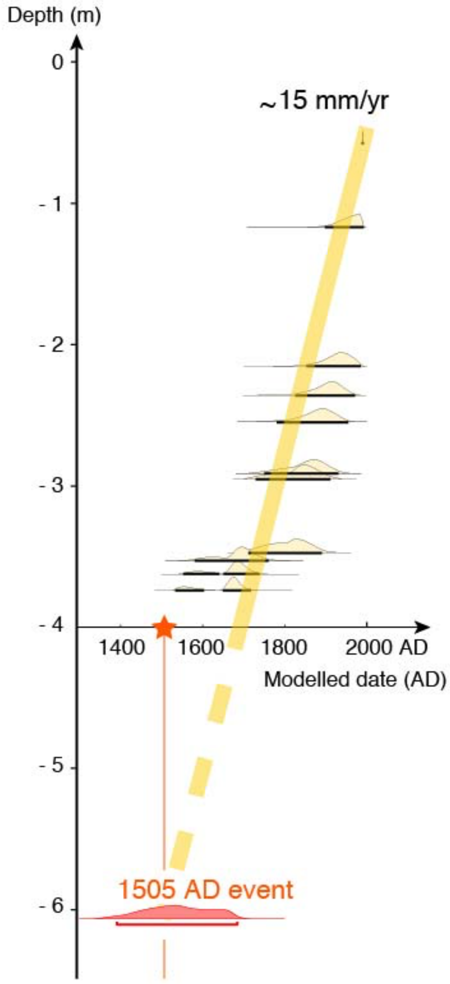

Figure S1: Ages-depth profile within U1 and U2 constrained by the bayesian model detailed in the text. The posterior ages PDF of the detrital charcoals are represented in yellow. The red Pdf corresponds to the earthquake horizon, assuming that the main collapse wedge was triggered by a local earthquake. As a matter of comparison, the thick plain line in yellow illustrates a sedimentation rate at ~ 1.5cm/yr since1691. Figure generated with Adobe illustrator CS6 (http://www.adobe.com/fr/products/illustrator.html).

Table S1

AMS Radiocarbon (^{14}C) Dates From Detrital Charcoals Collected From The Sukhetal Trench

Unit [a]	Sample number	Measured Radiocabon Age	$\delta^{13}C$ Value	Individually Calibrated Ages (Calendar, 2σ)[b]
U1	SUK18-77	114.11 +/-0.43 pMC	-26.6 o/oo	1990-1993 CE or 1957-1958 CE
U1	SUK18-55	110 +/- 30 BP	-25.7 o/oo	1802-1938 CE
U1	SUK18-58	340 +/- 30 BP	-26.8 o/oo	1470-1640 CE
U1	SUK18-46	80 +/- 30 BP	-25.1 o/oo	1690-1926 CE
U1	SUK18-59	120 +/- 30 BP	-25.3 o/oo	1678-1940 CE
U1	SUK18-45	380 +/- 30 BP	-26.8 o/oo	1445-1632 CE
U1	SUK18-60	90 +/- 30 BP	-27 o/oo	1684-1928 CE
U1	SUK18-30	140 +/- 30 BP	-26.3 o/oo	1669 -1944 CE
U2	SUK18-51	320 +/- 30 BP	-26.4 o/oo	1482-1646 CE
U2	SUK18-72	320 +/- 30 BP	-25.0 o/oo	1482-1646 CE
U2	SUK18-73	190 +/- 30 BP	-26.5 o/oo	1648-Post 1950 CE
CW1	SUK18-20	200b+/-30 BP	-24.3 o/oo	1646-Post 1950 CE
CW1	SUK18-19	380 +/-30 BP	-25.4 o/oo	1445-1632 CE
CW1	SUK18-21	280 +/- 30 BP	-25.5 o/oo	1438-1795 CE
CW2	SUK18-18	790 +/-40 BP	NA	1169-1280 CE
CW2	SUK18-78	550 +/- 30 BP	NA	1311-1434 CE
CW2	SUK18-54	4290 +/- 50 BP	-26.8 o/oo	3028-2705 BCE

[a] See trench log for stratigraphic unit designations. [b] Minima and maxima pre-bomb calendric dates were calibrated using the atmostpheric calibration curve IntCal13 for the Northern Hemisphere [Reimer et al., 2013] and Post-bomb were calibrated using IntCal13+NHZ3.
